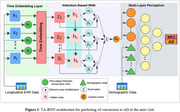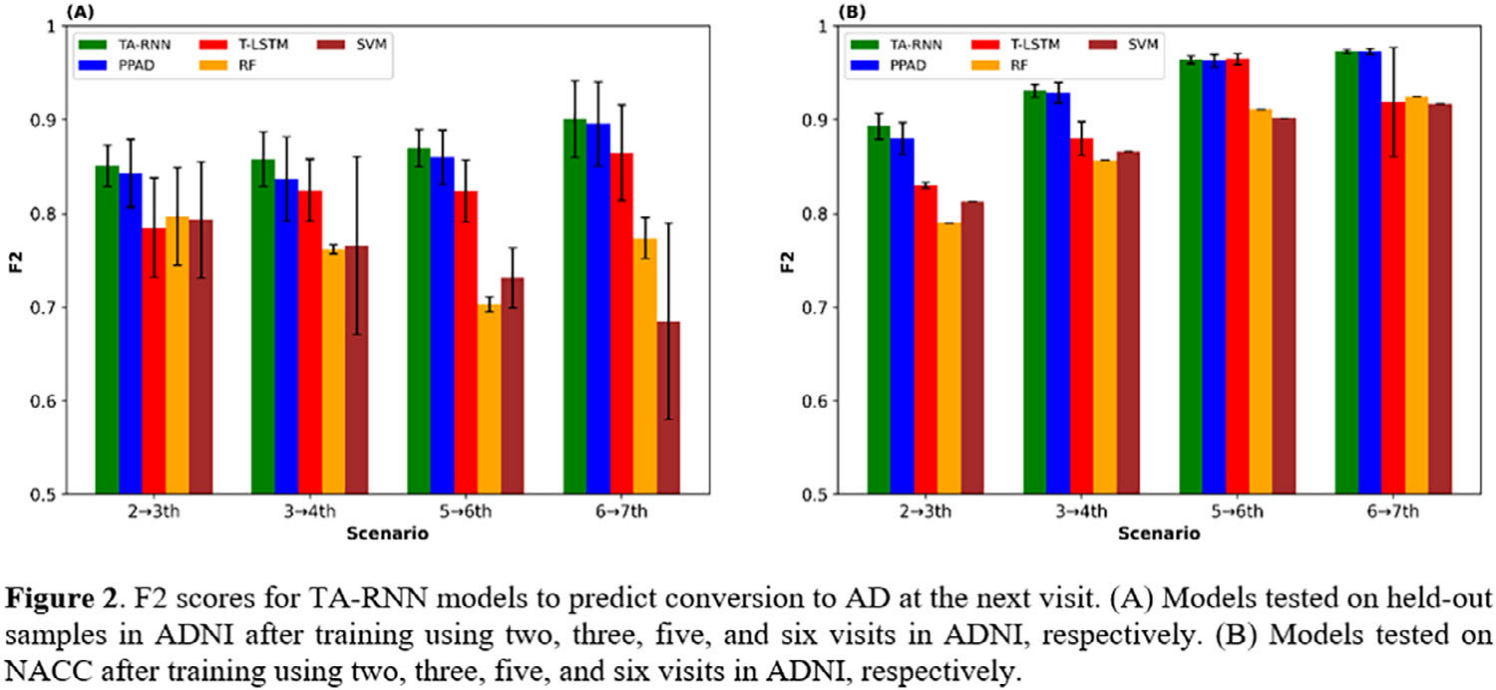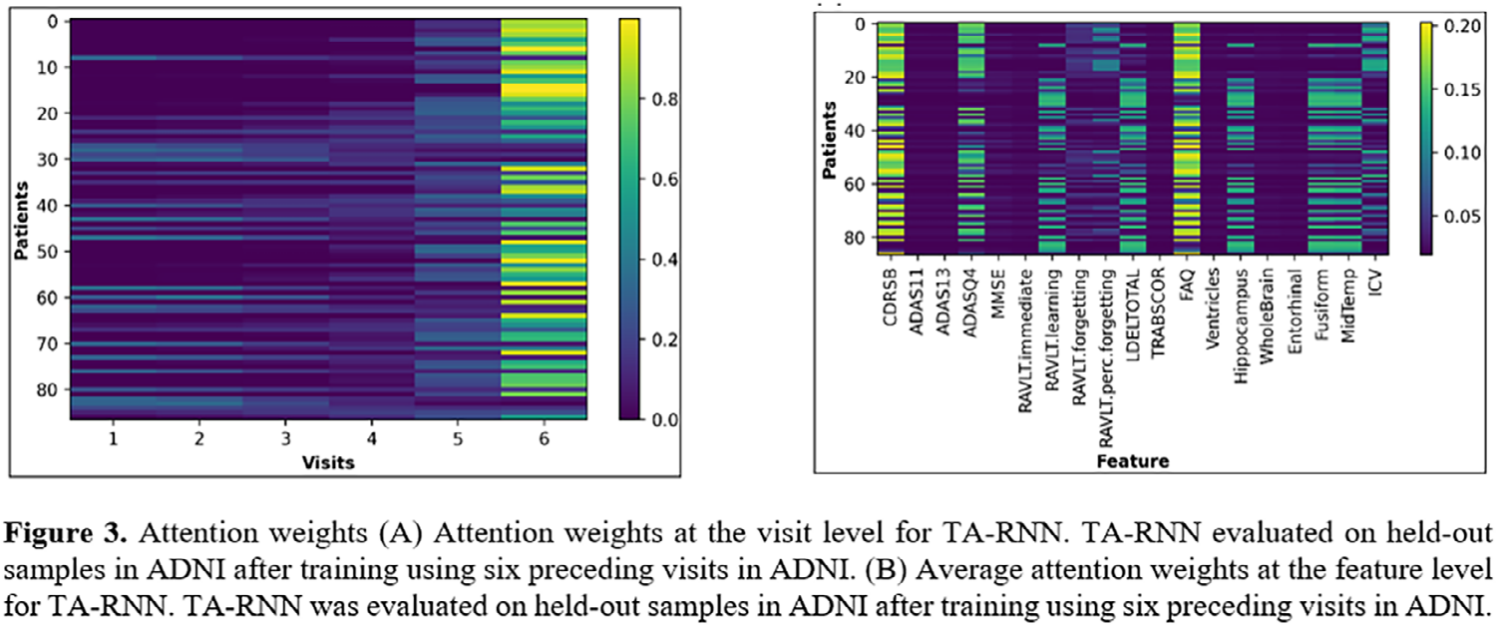# TA‐RNN: an Attention‐based Time‐aware Recurrent Neural Network Architecture to Predict Progression of Alzheimer’s Disease

**DOI:** 10.1002/alz.089010

**Published:** 2025-01-03

**Authors:** Mohammad Al Olaimat, Serdar Bozdag, Fahad Saeed, Alzheimer’s Disease Neuroimaging Initiative

**Affiliations:** ^1^ University of North Texas, Denton, TX USA; ^2^ BioDiscovery Institute / University of North Texas, Denton, TX USA; ^3^ Florida International University, Miami, FL USA

## Abstract

**Background:**

Alzheimer’s Disease (AD) is a widespread neurodegenerative disease with Mild Cognitive Impairment (MCI) acting as an interim phase between normal cognitive state and AD. The irreversible nature of AD and the difficulty in early prediction present significant challenges for patients, caregivers, and the healthcare sector. Deep learning (DL) methods such as Recurrent Neural Networks (RNN) have been utilized to analyze Electronic Health Records (EHR) to model disease progression and predict diagnosis. However, these models do not address some inherent irregularities in EHR data such as irregular time intervals between clinical visits. Furthermore, most DL models are not interpretable. To address these issues, we developed a novel DL architecture called Time‐Aware RNN (TA‐RNN) to predict MCI to AD conversion at the next clinical visit.

**Method:**

TA‐RNN comprises of a time embedding layer, attention‐based RNN, and prediction layer based on multi‐layer perceptron (MLP) (Figure 1). For interpretability, a dual‐level attention mechanism within the RNN identifies significant visits and features impacting predictions. TA‐RNN addresses irregular time intervals by incorporating time embedding into longitudinal cognitive and neuroimaging data based on attention weights to create a patient embedding. The MLP, trained on demographic data and the patient embedding predicts AD conversion. TA‐RNN was evaluated on Alzheimer’s Disease Neuroimaging Initiative (ADNI) and National Alzheimer’s Coordinating Center (NACC) datasets based on F2 score and sensitivity.

**Result:**

Multiple TA‐RNN models were trained with two, three, five, or six visits to predict the diagnosis at the next visit. In one setup, the models were trained and tested on ADNI. In another setup, the models were trained on the entire ADNI dataset and evaluated on the entire NACC dataset. The results indicated superior performance of TA‐RNN compared to state‐of‐the‐art (SOTA) and baseline approaches for both setups (Figure 2A and 2B). Based on attention weights, we also highlighted significant visits (Figure 3A) and features (Figure 3B) and observed that CDRSB and FAQ features and the most recent visit had highest influence in predictions.

**Conclusion:**

We propose TA‐RNN, an interpretable model to predict MCI to AD conversion while handling irregular time intervals. TA‐RNN outperformed SOTA and baseline methods in multiple experiments.